# HEIDI: an experiment-management platform enabling high-throughput fragment and compound screening

**DOI:** 10.1107/S2059798324002833

**Published:** 2024-04-12

**Authors:** A. Metz, D. P. Stegmann, E. H. Panepucci, S. Buehlmann, C.-Y. Huang, K. E. McAuley, M. Wang, J. A. Wojdyla, M. E. Sharpe, K. M. L. Smith

**Affiliations:** a Swiss Light Source, Paul Scherrer Institute, 5232 Villigen PSI, Switzerland; Lund University, Sweden

**Keywords:** HEIDI, *FFCS* software suite, data management, fragment screening, high-throughput crystallography, software

## Abstract

The Swiss Light Source facilitates fragment-based drug-discovery campaigns for academic and industrial users through the *Fast Fragment and Compound Screening* (*FFCS*) software suite. This framework is further enriched by the option to utilize the *Smart Digital User* (*SDU*) software for automated data collection across the PXI, PXII and PXIII beamlines. In this work, the newly developed HEIDI webpage (https://heidi.psi.ch) is introduced: a platform crafted using state-of-the-art software architecture and web technologies for sample management of rotational data experiments. The HEIDI webpage features a data-review tab for enhanced result visualization and provides programmatic access through a representational state transfer application programming interface (REST API). The migration of the local *FFCS* MongoDB instance to the cloud is highlighted and detailed. This transition ensures secure, encrypted and consistently accessible data through a robust and reliable REST API tailored for the *FFCS* software suite. Collectively, these advancements not only significantly elevate the user experience, but also pave the way for future expansions and improvements in the capabilities of the system.

## Abstract 2

The HEIDI experiment-management platform supports high-throughput fragment screening and facilitates structure-based research, design and drug discovery by both academic and industrial users at the Swiss Light Source crystallization facility and macromolecular crystallography beamlines. Collectively, the software solutions streamline sample preparation, beamline data acquisition and data processing, and facilitate fully automated experiments.

## 1. Introduction

Fragment-based drug discovery (FBDD) has been contributing constructively to early lead discovery for over two decades (Erlanson *et al.*, 2016 ▸; Woodhead *et al.*, 2024 ▸). With the first large successes of FBDD leading to the development and approval of the cancer drug vemurafenib in 2011 (Bollag *et al.*, 2010 ▸; Tsai *et al.*, 2008 ▸), FBDD has established itself as a valid, orthogonal strategy in academic and industrial drug development. Currently, seven drugs derived from FBDD have been approved by the US Food and Drug Administration, with over 50 fragment-derived compounds currently in clinical trials (Woodhead *et al.*, 2024 ▸). The idea of probing smaller and therefore more promiscuously binding ligands for interaction rather than target modulation was coined into the so-called ‘rule of three’ by Congreve *et al.* (2003 ▸), and is still applied with small variations and updates specific for the actually utilized biophysical method, for example NMR, surface plasmon resonance or thermal shift assay. A particularly interesting method to identify fragment binders is presented in the use of X-ray crystallography. The unambiguous hit identification, in combination with insight into the binding pose and possible directions for ligand growth, obtained from the three-dimensional structural information provides a unique starting point for pre-lead development and later facilitates structure-guided drug development. Recent improvements in data-collection speed, quality and general beamline automation at synchrotrons (Winter & McAuley, 2011 ▸; Smith *et al.*, 2023 ▸; Martiel *et al.*, 2020 ▸; O’Hea *et al.*, 2018 ▸; Casanas *et al.*, 2016 ▸; Oscarsson *et al.*, 2019 ▸; Schiebel *et al.*, 2016 ▸; Bowler *et al.*, 2015 ▸) have increased the feasibility of this method. In recent years, data-management systems have proven to be critical for handling the influx of data that is common in high-throughput crystallography and fragment-screening experiments. Some notable examples of laboratory information-management systems that have arisen from crystallization facilities and universities are CRIMS (Cornaciu *et al.*, 2021 ▸) and IceBear (Daniel *et al.*, 2021 ▸). These laboratory-based systems are then able to interact with well established synchrotron ISPyB-based systems, including SynchWeb (Fisher *et al.*, 2015 ▸) and ExiGUI (Delagenière *et al.*, 2011 ▸), with an API for orchestrating sample logistics and transfer of raw and processed data. To cope with the large number of data sets to be curated and processed in fragment-screening campaigns, the *FragMAX* app was developed to allow seamless post-experimental processing and analysis (Lima *et al.*, 2020 ▸). Software developments that allow robust, automated data reduction and even pre-refinement and hit identification can assist operators further in speeding up data assessment.

In combination, these advancements now allow a user to routinely collect 20–30 diffraction data sets per hour, resulting in substantial throughput during the actual experiment and subsequent data assessment. With a greater abundance of beamtime at synchrotrons, the bottleneck of this technique has become the sample-preparation step. After establishing robust, reproducible crystal growth, several hundreds to thousands of crystals must be incubated in a corresponding fragment solution. To speed up this meticulous manual labour, dedicated fragment-screening facilities have been established at several synchrotrons to assist users in this task (Kaminski *et al.*, 2022 ▸; Cipriani *et al.*, 2012 ▸; Lima *et al.*, 2020 ▸; Douangamath *et al.*, 2021 ▸; Wollenhaupt *et al.*, 2020 ▸). These facilities use different approaches to assist the ligand transfer and soaking process, to minimize handling steps and the corresponding strain put on the crystals, and to avoid errors through automated bookkeeping for each individual crystal.

## 2. Fast fragment screening and automation at the Swiss Light Source

Our Fast Fragment and Compound Screening (FFCS) facility at the Swiss Light Source (SLS) has effectively aided scientists across many campaigns (Bedi *et al.*, 2020 ▸; Sutanto *et al.*, 2021 ▸; Huang *et al.*, 2024 ▸). The FFCS utilizes a Formulatrix Rock Imager to store and image crystals grown in SWISSCI 3 Lens plates (MRC3). The standard procedure to carry out soaking experiments with FFCS uses offset targeting with an Echo (Beckman) contactless liquid-transfer robot that delivers pre-dissolved fragments or other ligands directly into the crystallization droplets (Collins *et al.*, 2017 ▸). After the desired incubation time, a shifter robot (Oxford Lab Technologies) assists the user with semi-automated crystal harvesting by presenting the right well to fish from under the microscope to the experimenter, removing the necessity to open every crystal well individually (Wright *et al.*, 2021 ▸).

**Figure 1:**
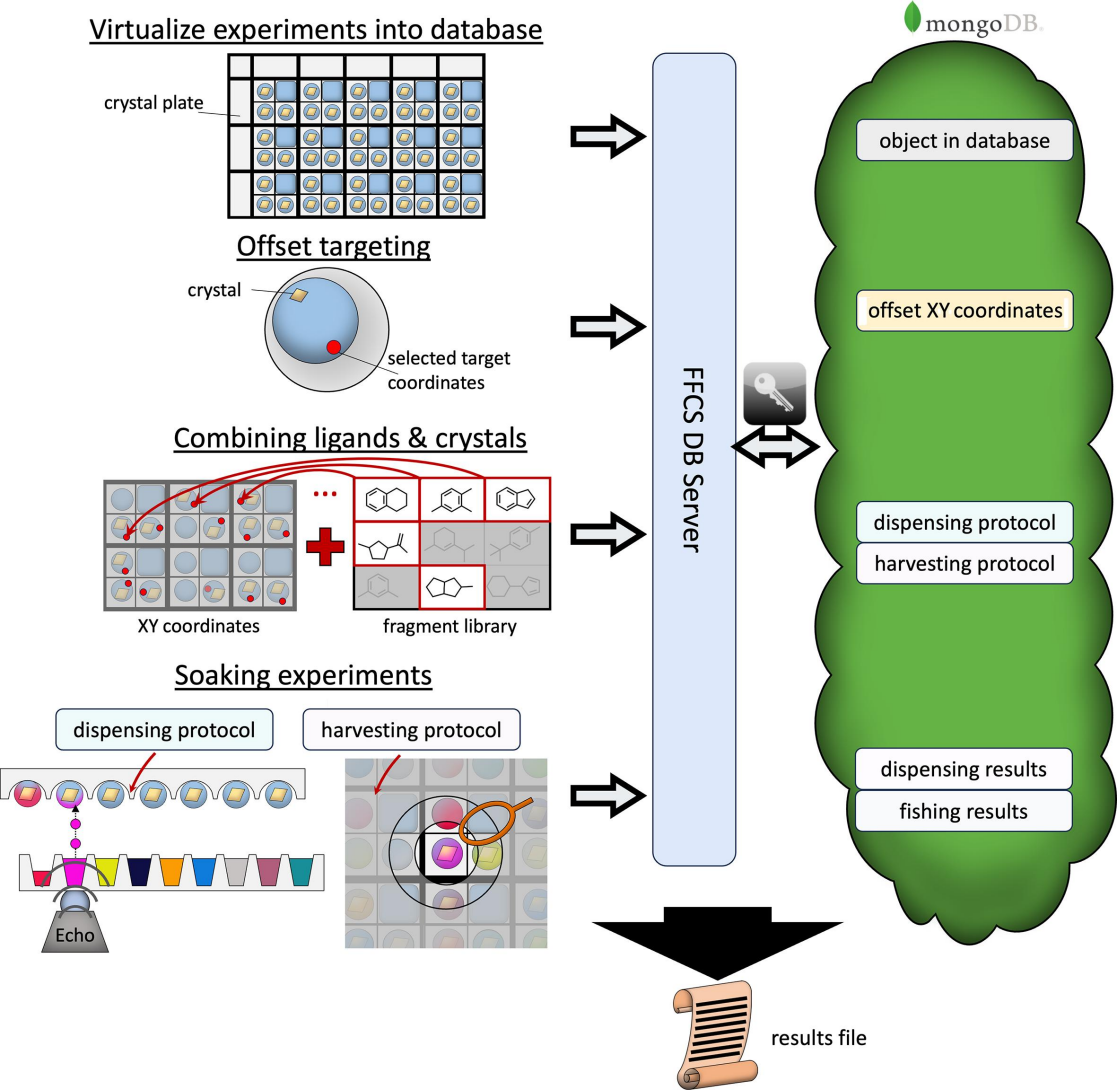
User workflow for campaigns with the *FFCS* software. A typical FFCS experiment begins with the creation of a project ID in the MongoDB Atlas. As a first step, each crystal plate designated to be used in a fragment-screening experiment is recreated virtually as an object in the database to facilitate access to the corresponding images of each individual crystal well taken with the Rock Imager. These images are necessary for the second step, where they are visually assessed by the user to define the *XY* target coordinates for the subsequent ligand-solution transfer. This is a crucial step to prevent damage to the crystals during ligand transfer and facilitates crystal assessment, selection and targeting in one quick step. Selected wells and target coordinates are stored in the database and used to create input files for the Echo dispenser and the shifter device later. Timestamps for soaking duration are created in the database by a small GUI called *soakMe* to keep track of the exact incubation times for each individual crystal. During the shifter-assisted crystal harvesting, parameters such as harvesting status, observed crystal quality, puck name and puck location, and the time required to harvest each crystal are recorded and stored in the database via the *shiftMe* GUI. Finally, a complete experimental report can be extracted from the *FFCS* GUI, which includes all of the previously collected data in an .xls file format, and a data-collection spreadsheet for data collection at SLS can be derived from it.

The robust orchestration of all of these steps and machines is facilitated and expedited by the *FFCS* GUI management software (Kaminski *et al.*, 2022 ▸). This Python-based graphical user interface (GUI) connects all of the experimental steps and keeps track of the current status of each individual crystal at every stage of the experiment (Fig. 1 ▸). The currently ongoing upgrade of the SLS to a fourth-generation synchrotron and the requirement for secure remote access and assessment of larger data rates necessitates the development of an improved data-management system. To address this requirement, the HEIDI experiment-management platform was developed, which serves as a sophisticated frontend interface for the planning, co­ordination and evaluation of data collection. This platform supports both standard rotational and more complex MX experiments, streamlining the experimental workflow. In the future, HEIDI will comprise the results of FFCS campaigns to allow convenient, remote access to the individual data sets, results and potential follow-up experiments.

## 3. HEIDI software ecosystem for data management

**Figure 2:**
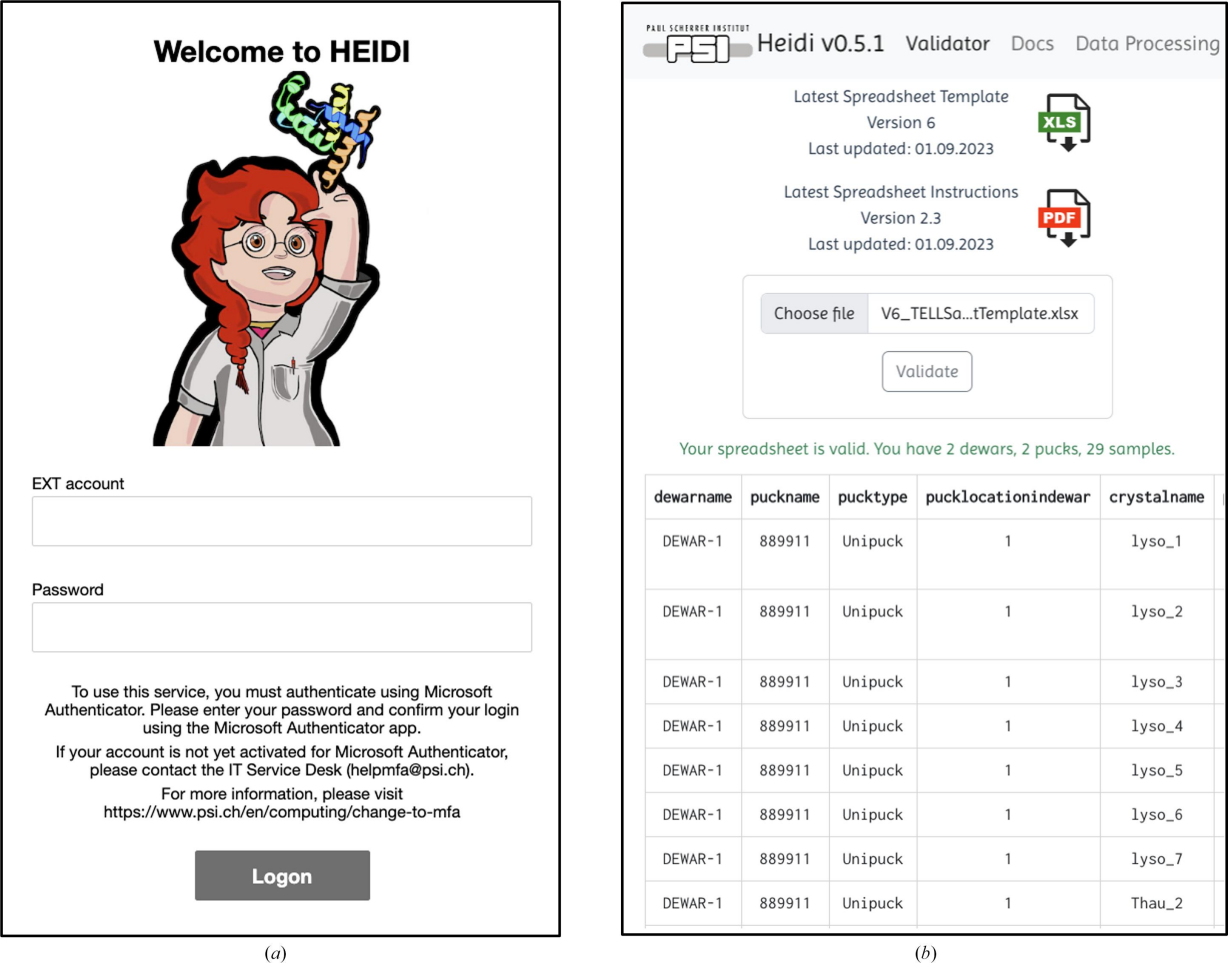
User login and spreadsheet validation. (*a*) Users first login with their PSI user account and must then authenticate their login attempt with the Microsoft Authenticator application. (*b*) Once users have access to HEIDI they can then navigate to the Validator tab for spreadsheet download and validation.

The HEIDI webpage (https://heidi.psi.ch) serves as a robust platform for users to prepare and analyse the results of experiments performed at the SLS. The process begins with the sample spreadsheet, which can be crafted manually, generated via the users’ preferred sample-management software or created at SLS through the *FFCS* GUI. It contains essential information for sample mounting, data acquisition and data processing. Users can easily validate sample spreadsheets prior to an experiment, view a live stream of their data-processing results during an experiment, and visualize processing results post-experiment, all from the internet. The HEIDI webpage utilizes multi-factor authentication (MFA); therefore, for a user to gain access they will first need to login with their Paul Scherrer Institute (PSI) user account and then authenticate their login attempt with the Microsoft Authenticator application (Fig. 2 ▸ *a*).

Once logged in, a user can download the latest sample spreadsheet and instructions directly from the web. They can also upload their completed sample spreadsheet to validate and visually inspect their sample inputs before their experiment (Fig. 2 ▸ *b*).

**Figure 3:**
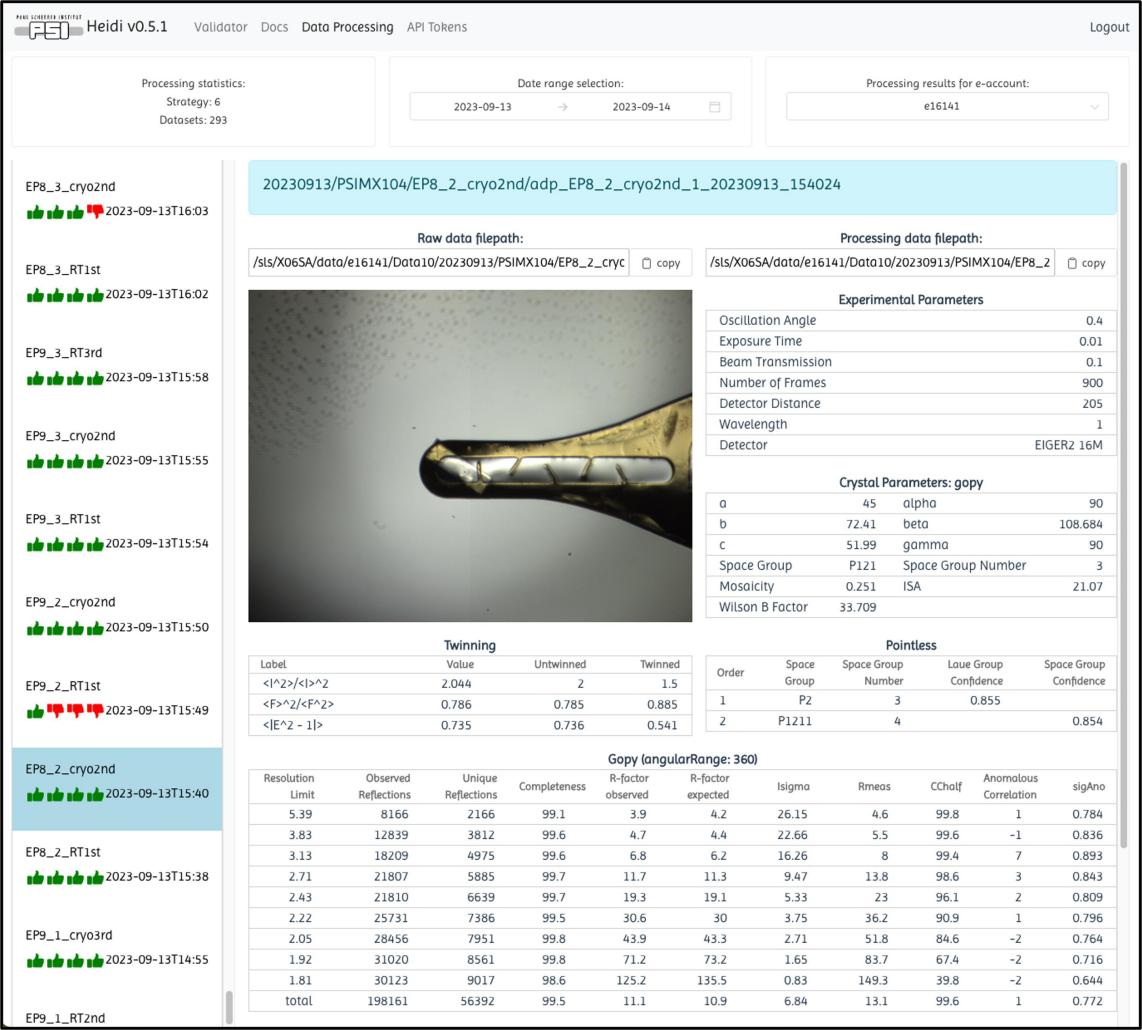
HEIDI webpage data-processing results view. At the top left, users can see a brief overview of the number of data sets collected for the date range selected in the middle widget and the experiment account selected in the top-right drop-down menu.

Users who have utilized the *FFCS* software suite at the SLS will have their validated spreadsheet automatically generated and locatable in their ~/Data10/FFCS folder. HEIDI integrates pre-existing data-acquisition, data-processing and automation software into its data-management ecosystem (Wojdyla *et al.*, 2018 ▸; Martiel *et al.*, 2020 ▸; Smith *et al.*, 2023 ▸). This integration allows users of HEIDI to leverage the same data models as our data-acquisition and processing software, ensuring a seamless user experience of the beamline software and when validating their sample spreadsheet. Importantly, users will only have access to experiments linked to their PSI user accounts, ensuring strict confidentiality. This access is designated by the main proposer of each experiment account using a permissions group (p-group) through the Digital User Office (https://duo.psi.ch). The main proposers have complete control over data access by dynamically adding or removing PSI user accounts from their experiment proposal p-group. Users with multiple experiment accounts can select a specific account and the desired date ranges from dropdown and calendar widgets, respectively, to view the corresponding processing results in HEIDI (Fig. 3 ▸). HEIDI provides views of standard rotational data-processing results from *autoPROC* (Vonrhein *et al.*, 2011 ▸), *xia*2*dials* (Winter *et al.*, 2022 ▸) and the in-house processing pipeline *gopy*, which is an improved version of the *go.com* script (Wojdyla *et al.*, 2018 ▸) implemented in Python. Academic users and members of the Global Phasing Consortium with active licences are welcome to use *autoPROC* at the SLS for their data-processing needs. In the left-hand scroll bar, green thumbs-up and red thumbs-down icons represent the progress through our four-step data-processing sequence. Step 1 comprises a step called initialization, which involves confirming that the data expected to be collected from the detector have finished being written to the filesystem and are available for processing to begin. Step 2 comprises fast indexing with 33% of the data. Step 3 comprises fast integration with 66% of the collected data to provide preliminary data-quality statistics. Finally, Step 4 comprises the complete processing of the data set using one of the three available software options. Users can also conveniently copy the file-path locations of their raw and processed data using dedicated buttons, facilitating data-set reprocessing on the beamline cluster and data transfer to their home laboratory.

**Figure 4:**
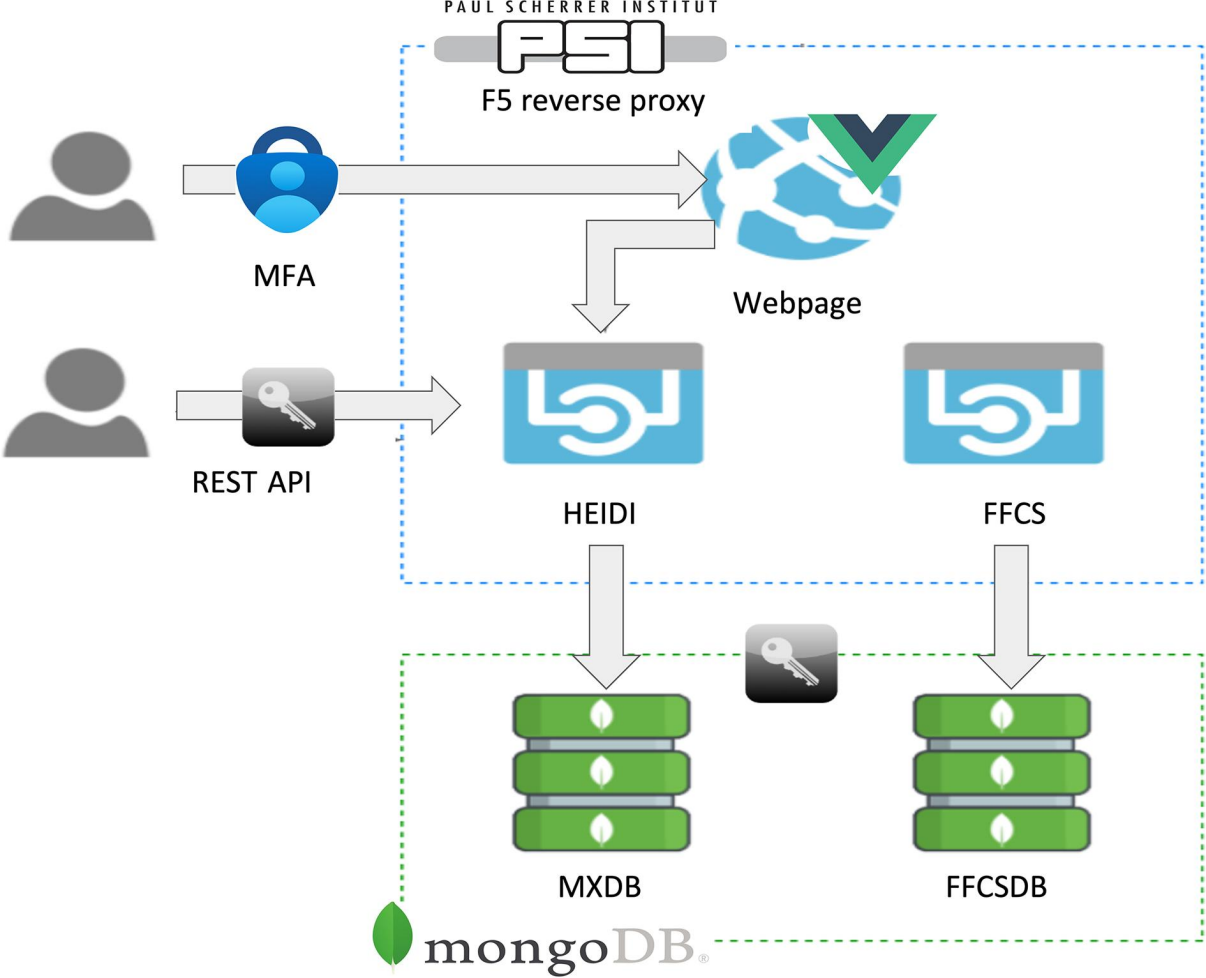
HEIDI dual-access architecture diagram. Users can login to the HEIDI webpage using MFA with their PSI user account and the Microsoft Authenticator mobile application. This provides access to the HEIDI application. Alternatively, users have the option to access the HEIDI application REST API via a secure UUID4 token generated directly from the HEIDI webpage. The *FFCS* applications are currently only available from within the crystallization facility.

HEIDI was designed with a dual-access architecture to engage a wider community of users, offering both a user-friendly webpage and a representational state transfer application programming interface (REST API) for programmatic access (Fig. 4 ▸). Providing a REST API access further enhances the utility of HEIDI, allowing users to bypass the webpage for data retrieval.

**Figure 5:**
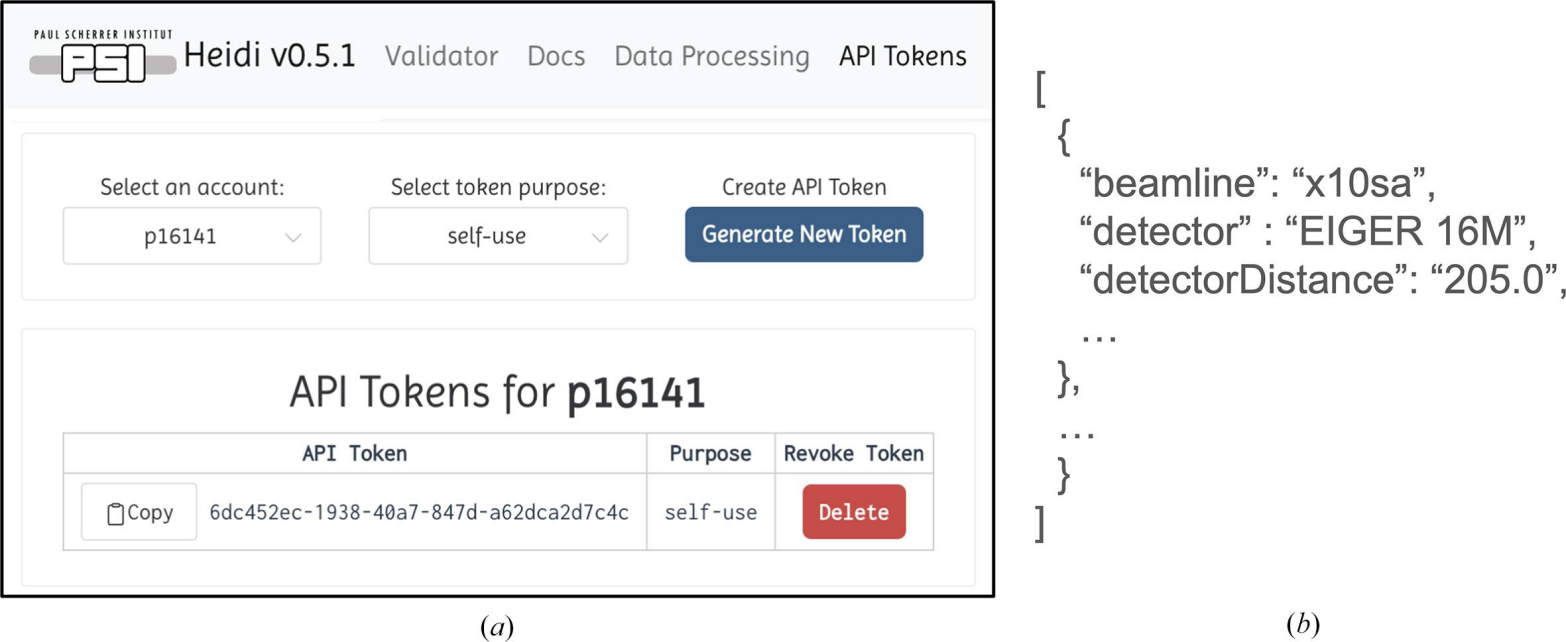
HEIDI token management and REST API usage. (*a*) Token generation and management. Users can select the account for which to create a token, select a purpose for the token from the options self-use, CRIMS and LIMS, and generate a new UUID4 token. Users then have a dedicated button to copy the API token to their clipboard. Users can also choose to delete a token and will be prompted a second time ‘are you sure you want to delete?’; the token will only be deleted after confirmation. (*b*) These panels show two examples of command-line requests to the REST API and the returned processing data as a list of JSON documents.

To enable programmatic data access, users must generate a unique UUID4 (Leach *et al.*, 2005 ▸) token via the API Tokens tab on the HEIDI webpage. A dropdown menu allows the selection of an experiment account, for which a new token can be generated with a user-specified purpose (Fig. 5 ▸ *a*). Users can create and delete as many of these tokens as desired. Clicking a token’s delete button prompts a confirmation, and once deleted the action is final and the token cannot be recreated. This token management provides flexibility for users who prefer programmatic access to their data. The REST API provides information in JSON format, and processing results are returned as a list of JSON objects (Fig. 5 ▸ *b*).

**Figure 6:**
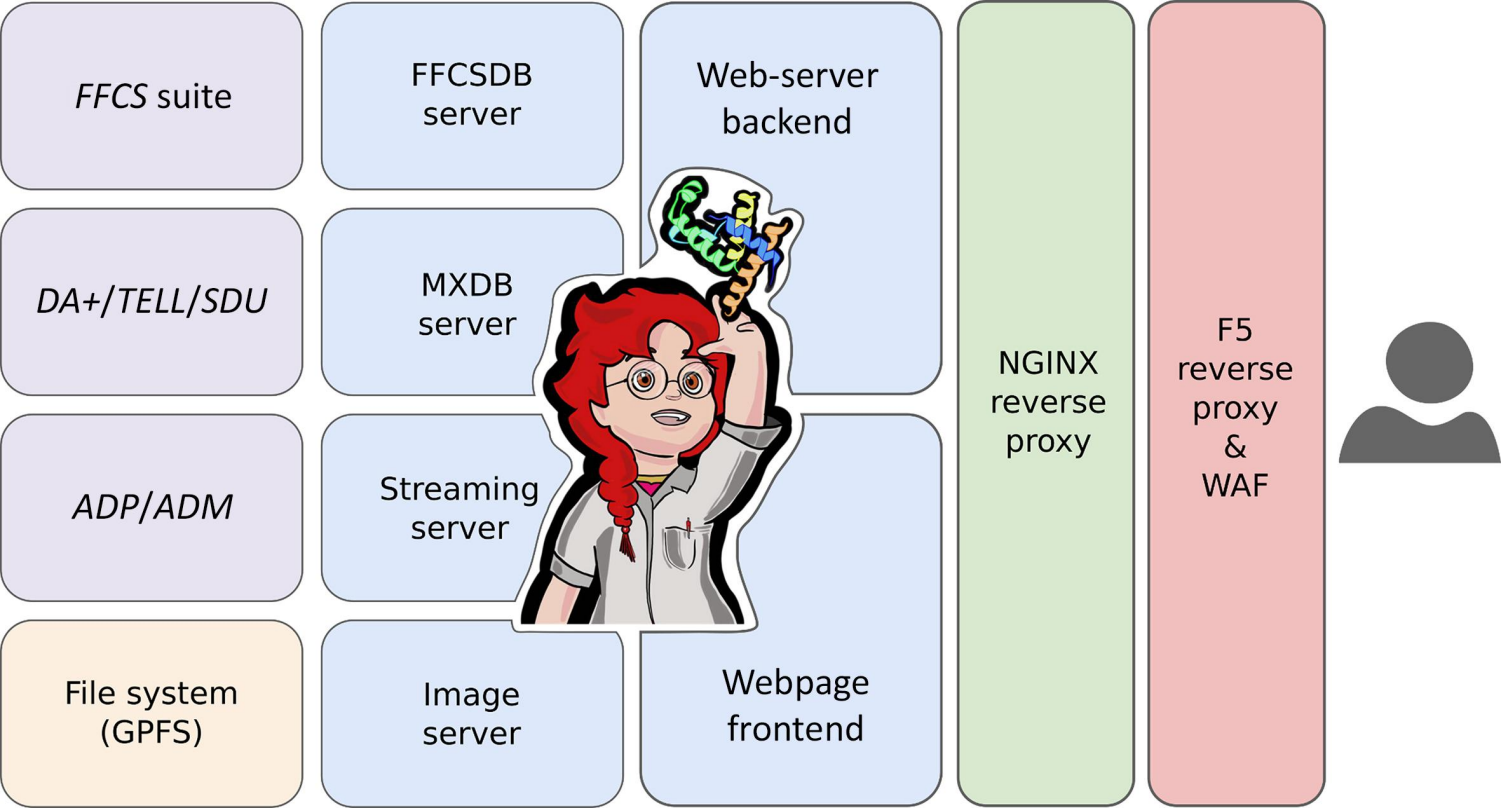
HEIDI microservice architecture diagram. Pre-existing fragment-screening (*FFCS* suite), data-acquisition (*DA*+), sample-mounting (*TELL*), fully automated data-collection (*SDU*), data-processing (*ADP*) and data-merging (*ADM*) software are shown in purple. The central General Parallel File System (GPFS) is shown in orange. The various HEIDI software are shown in blue (https://github.com/HeidiProject). The NGINX reverse proxy that sits in front and orchestrates communication to HEIDI microservices is shown in green. The BIG-IP F5 reverse proxy, web application firewall (WAF) and authentication server located between HEIDI and the internet are shown in red.

To ensure a sustainable and easily maintainable software infrastructure, HEIDI adopts a microservices architecture (Fig. 6 ▸). Components include an NGINX reverse proxy, a Python Flask backend, a Vue3 frontend, a FastAPI image server, a Python Flask database server and a dedicated FastAPI database streaming service. This architecture facilitates scalability and streamlines software-development efforts, allowing multiple technologies to work together seamlessly. Thereby, the current status of HEIDI, prior to the finalization of the fourth-generation SLS upgrades, is a solid foundation for future development.

## 4. Data-management integration of *FFCS* into HEIDI

The *FFCS* suite is in continuous use and has recently undergone substantial data-management technology upgrades to sustainably support its functionality for users. A key improvement is migrating the FFCS database to a cloud-based infrastructure. This was performed without interruption to user operations and without modification of the user GUIs and workflows. This move ensures high reliability and availability, strong security measures and continuous 24/7 support. The database is now hosted on a MongoDB Atlas v5.0 Microsoft Azure cloud solution configured as an M10 cluster, utilizing encryption at storage and during transit, along with strict IP access controls. To streamline database interactions, a FastAPI server, *ffcs_db_server*, was developed, integrated and dockerized for enhanced portability. Pydantic models (https://github.com/pydantic/pydantic) are employed for data validation, and a dedicated Python client, *ffcs_db_client*, facilitates the efficient management of requests to the FastAPI server. The database interactions and models are consistent with those published previously (Kaminski *et al.*, 2022 ▸), featuring optimized queries and indexing tailored to historical usage patterns for improved performance.

## 5. Conclusion and outlook

The new internet-accessible HEIDI webpage enables real-time tracking of data-processing results, offering a dynamic and user-focused approach to project management. This is complemented by the successful refactoring of the previous FFCS database system into a secure, highly available and performant cloud-based solution, marking a significant step in enhancing the reliability and security of the *FFCS* software suite. The source code for the repositories mentioned from the HEIDI project, including the new FFCS database server and client, are openly available in GitHub with a permissive BSD-3 licence at https://github.com/HeidiProject.

Looking ahead, the focus shifts towards future projects aimed at expanding the capabilities of the *FFCS* suite. One such objective involves providing post-refinement information, with a specific emphasis on presenting a comprehensive list of potential ligand hits and their poses derived from difference electron densities. Furthermore, the roadmap includes an extension of the functionality of the HEIDI platform to incorporate FFCS information and results seamlessly. We also aim to incorporate an editing tool for users to be able to readily modify uploaded spreadsheets for future experiments, a link to SciCat for data archiving, retrieval and DOI generation (https://doi.psi.ch), and the visualization of more complex experiments, such as multi-orientation data collection, serial rotational and serial still images. Eventually, we plan to implement and interface HEIDI with computer-aided drug-discovery workflows, utilizing the rich data generated by FFCS, to enhance our research capabilities in drug development. These forward-looking initiatives underscore the commitment to continuous improvement, innovation and the advancement of fragment-based drug-discovery methodologies at the SLS.
